# Risk factors for repeated general anesthesia for dental treatment of adult patients with intellectual and/or physical disabilities

**DOI:** 10.1007/s00784-021-04142-w

**Published:** 2021-08-25

**Authors:** Mona Shaghayegh Maes, Philipp Kanzow, Jana Biermann, Andreas Leha, Valentina Hrasky, Annette Wiegand

**Affiliations:** 1 Department of Preventive Dentistry, Periodontology and Cariology, University Medical Center Göttingen, Robert-Koch-Str. 40, D-37075 Göttingen, Germany; 2 Department of Medical Statistics, University Medical Center Göttingen, Göttingen, Germany

**Keywords:** Disability, General anesthesia, Restoration, Extraction, Kaplan–Meier statistics

## Abstract

**Aim:**

Repeated dental treatment of patients with intellectual and/or physical disabilities under general anesthesia (GA) often becomes necessary. This study aimed to identify potential risk factors predictive of repeated dental treatment under general anesthesia.

**Materials and methods:**

Data of adult patients with intellectual and/or physical disabilities receiving dental treatment under GA within a time period of 7 years were analyzed (*n* = 203, mean age: 41.0 ± 14.9 years). All patients received comprehensive dental treatment (professional tooth cleaning, periodontal therapy, composite restorations, and/or extractions); patients receiving extractions only for emergency dental care were not included as a second intervention for restorative treatment often followed. Demographic, anamnestic, oral health, and treatment factors were obtained from dental records. Duration of intervals without dental treatment under GA was assessed using Kaplan–Meier statistics. Potential predictive factors were tested using univariate and multivariate cox regression analyses.

**Results:**

Thirty-five patients (17.2%) received a second and five patients (2.5%) a third dental treatment under GA during that period. In the univariate analysis, patients’ age, living situation, and nutrition were associated with repeated GA. In the multivariate Cox regression analysis, only nutrition remained significant. Risk for repeated treatment increased if patients were tube-fed (HR: 7.54, *p* = 0.001) or received pureed/liquid food (HR: 4.32, *p* = 0.007) compared to nutrition without limitation.

**Conclusion:**

In adult patients with intellectual and/or physical disabilities, nutrition affects the risk for repeated dental treatment under GA.

**Clinical relevance:**

Identification of risk factors making repeated dental treatment under GA of patients with intellectual and/or physical disabilities more likely is essential to adjust preventive measures.

## Introduction

Adult patients with severe intellectual disabilities are often affected by caries and periodontal diseases requiring extensive dental treatment [1]. Due to a low level of cooperation and with regard to the quantity and intended quality of oral care, operative interventions under general anesthesia (GA) often become necessary.

Preoperatively, treatment need of these patients is often underestimated by both caregivers and dentists [2]. Restorations and extractions are more often done under GA than endodontic treatment [3–6]. Especially in special needs patients, extractions are often preferred over restorative treatments, as they might reduce the need for repeated GA and thus associated postoperative risks. Nevertheless, dental treatment under GA improves the quality of life of patients with intellectual disabilities [7, 8].

As the abilities of dental professionals and caregivers to maintain the oral health status achieved under GA are limited by various factors, e.g., cooperation of the patient, lack of training of nursing staff, health insurances not covering costs for regular preventive treatment, repeated dental treatment under GA often becomes necessary. Recent long-term studies reported 25 to 27% of disabled adult patients to receive repeated dental treatment within a time period of 10 to 12 years [3, 5, 6].

However, clinical outcomes of dental treatment under GA of adult patients with intellectual and/or physical disabilities have been rarely investigated [4, 9], and there is no information on risk factors making repeated GA in adult special needs patients more likely. The aim of this study was to retrospectively evaluate the characteristics of special needs patients with regard to repeated treatment under GA. The null hypothesis was that demographic and anamnestic data as well as oral health were not associated with repeated dental treatment under GA.

## Materials and methods

This study was approved by the Ethics Committee of the University Medical Center Göttingen (15/1/18) and registered at ClinicalTrails.gov (NCT04407520). In this retrospective single-center study, all dental records of adult special needs patients receiving dental treatment (professional tooth cleaning, periodontal therapy, composite restorations, and/or extractions) under GA from January 2011 to December 2017 in the Department of Preventive Dentistry, Periodontology and Cariology of the University Medical Center Göttingen were analyzed. The inclusion criteria were as follows: (1) patients ≥ 18 years, (2) patients lacking cooperation due to intellectual and/or physical disabilities or other neurocognitive disorders, and (3) patients received at least one dental treatment under GA. Patients receiving extractions only for emergency dental care were not included as a second intervention for restorative treatment often followed.

Demographic/anamnestic data, information about oral health prior and after dental treatment under GA and about the extent of dental treatment under GA were obtained from dental records.

The following demographic/anamnestic data were considered: Age, gender, type of disability (intellectual, physical, combined intellectual and physical), presence of a legal guardian, living situation (alone, with family, care facility), nutrition (without restrictions, pureed/liquid food, feeding tube), oral hygiene (alone, with support, impossible), previous dental treatments under GA or sedation (yes/no), postoperative checkup within 3 months (yes/no), and average number of follow-up visits per year.

To assess dental health prior and after dental treatment, the number of decayed (D), missed (M), and filled (F) teeth (DMFT) and data on periodontal health were determined. Data on periodontal health were taken from a previous study, investigating a partially overlapping cohort [10]: periodontitis (yes/no; defined as more than 2 mm radiographically visible bone loss) and percent bone loss as a function of age.

### Statistical analysis

Kaplan–Meier estimate was used to measure time until second dental treatment under GA. Potential predictive factors were tested first using univariate Cox regression models and the likelihood ratio tests (*α* = 10%); the significant factors were then submitted to a multivariate Cox regression model (*α* = 5%). All analyses were performed with the statistic software R (version 3.6.1; www.r-project.org) using the package “survival” (version 2.44.1.1) for the time-to-event analyses.

## Results

A total of 203 patients (88 females, 43.3%), aged from 18 to 81 years (mean age: 41.0 ± 14.9 years), were included in the analysis. During the first GA, 1191 restorations and 576 extractions were performed. Thirty-five patients (17.2%, mean age at time of second dental treatment: 36.1 ± 13.1 years) received a second and five patients (2.5%, mean age at time of third treatment: 42.6 ± 16.4 years) a third dental treatment under GA. During these treatments, 98 (second GA) and 12 (third GA) restorations as well as 76 (second GA) and 4 (third GA) extractions were performed.

**Fig. 1:**
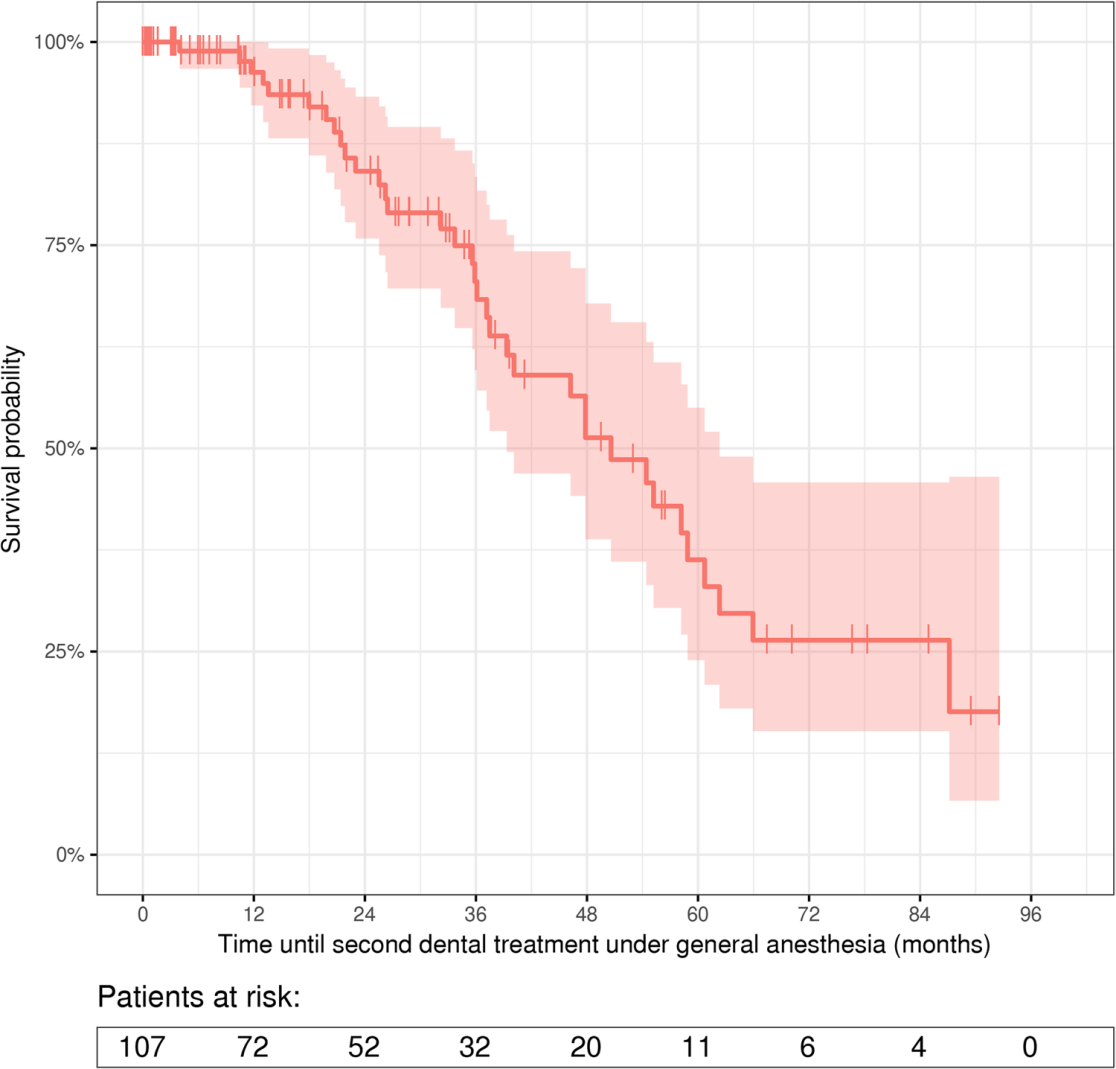
Kaplan–Meier graph for time until second dental treatment under general anesthesia. Ninety-six patients were excluded from the Kaplan–Meier analysis because of missing follow-up visits

Follow-up data after first treatment under GA was available for 107 patients. For these patients, Kaplan–Meier statistics was used to estimate time until second dental treatment under GA. The estimated probability for repeated dental treatment under GA amounted to 51.5% (95% CI: 39.0–68.0%) after 4 years (Fig. 1).

Variables tested for univariate association to the time for second treatment under GA are presented in Table 1. Age, living situation, and nutrition showed a significant univariate association to the time until second dental treatment under GA (*p* < 0.1).

**Table 1:** Demographic, anamnestic, and treatment factors of patients treated in general anesthesia and *p* values from likelihood ratio tests against the Null model

**Parameter**		***p* value**
**Age**	41.0 ± 14.9 years	**0.086**
**Gender**	Male (*n* = 115) Female (*n* = 88)	0.734
**Type of disability**	Intellectual (*n* = 68) Physical (*n* = 24) Both intellectual and physical (*n* = 111)	0.608
**Legal guardianship**	Yes (*n* = 186) No (*n* = 17)	0.784
**Living situation**	Care facility (*n* = 110) With family (*n* = 76) Alone (*n* = 10) Unknown (*n* = 7)	**0.033**
**Nutrition**	Without restrictions (*n* = 148) Pureed/liquid food (*n* = 31) Feeding tube (*n* = 19) Unknown (*n* = 5)	**0.046**
**Oral hygiene**	With support (*n* = 91) Alone (*n* = 81) Impossible (*n* = 26) Unknown (*n* = 5)	0.101
Previous dental treatments *	GA (*n* = 86) Sedation (*n* = 6)	0.442 0.899
**DMFT** D (before first GA) M (before first GA) M (after first GA) F (before first GA) F (after first GA)	18.4 ± 7.9 8.7 ± 6.4 5.7 ± 4.6 8.6 ± 6.3 3.9 ± 4.0 9.7 ± 5.2	0.971 0.624 0.704 0.950 0.689 0.807
**Periodontitis** (*n* = 197) **	Yes (*n* = 173) No (*n* = 24)	0.189
**Percent bone loss as a function of age** (*n* = 197) **	0.46 ± 0.48	0.978
**Recall appointments**	post-operative checkup within three months (n = 38) average number of follow-up visits per year (0.65 ± 1.31)	0.342 0.730

*p* values < 0.1 are printed in bold

*Multiple selections were possible

**For six patients, no full-mouth periapical radiographs were available

**Fig. 2:**
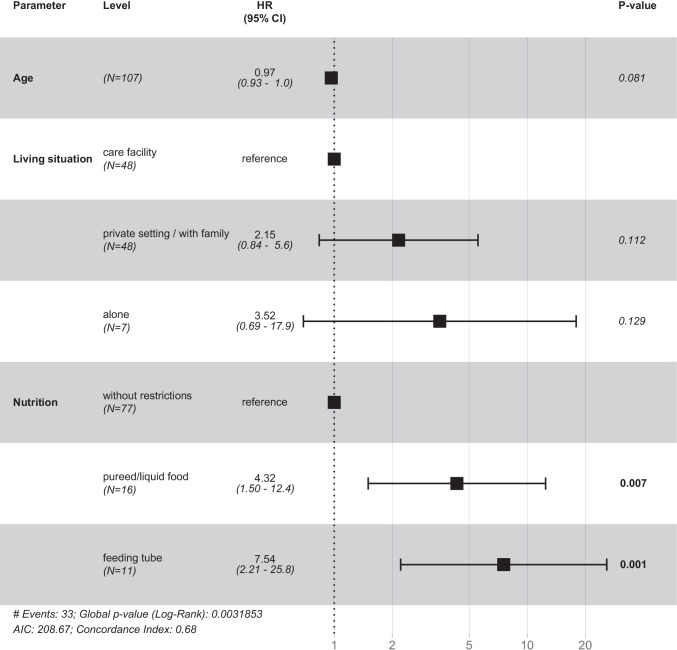
Forrest plot showing the results from the multivariate Cox regression analysis. Ninety-six patients were excluded from the Cox regression analysis because of missing follow-up visits. HR hazard ratio, 95% CI 95% confidence interval

The results of the multivariate Cox regression are presented in Fig. 2. Risk for repeated treatment increased if patients were tube-fed (HR: 7.54, *p* = 0.001) or received pureed/liquid food (HR: 4.32, *p* = 0.007) compared to nutrition without limitation.

## Discussion

In the present study, potential risk factors for repeated dental treatment under GA were identified, so that the null hypothesis was rejected.

The study population consisted of 203 patients, of which 17.2% received a second and 2.5% a third dental treatment under GA within the 7-year study period. This result is in line with the abovementioned studies reporting that about 25 to 27% of adult patients require repeated dental treatment under GA within 10 to 12 years [3, 5, 6]. The estimated probability for repeated dental treatment under GA amounted to 51.5% after 4 years. Previous studies reported the mean interval between dental treatments under GA of adult people with disabilities to amount to 3.1 to 3.5 years [3, 4].

Due to severe impairment, about half of the patients were not able to attend any routine dental recall appointment, so that these patients were excluded from the Kaplan–Meier statistics limiting the overall validity of the study. However, as our department is one of the very few specialized centers in the near surrounding, we do not assume that patients have received further dental treatments under GA elsewhere.

Another limitation of this study is the heterogeneous group of patients making standardization with respect to the impairment not possible. However, the vast majority of patients had a legal guardian, indicating a severe impairment due to intellectual and/or physical disabilities. The caries experience of our study population is higher compared to Germany’s adult population [11] and patients with intellectual disabilities in Germany [6, 8, 12] and Switzerland [3, 4]. However, the extent of treatment (number of restorations and extractions) well corresponds to previous studies [5, 6, 8].

Data were obtained retrospectively from paper-based records, relying on a proper and consistent documentation. As the vast majority of treatments under GA (about 95%) was performed by only two different operators, it was reasonable to assume that the quality of treatment and documentation is not too different.

We identified age, living situation, and nutrition to be associated with repeated GA in the univariate analyses, but only nutrition remained significant in the multivariate analysis. The need for pureed/liquid food or enteral nutrition indicated that patients have a higher level of disability compared to patients with normal nutrition. Previous studies showed that disabled patients receiving a liquid diet were shown to have a higher DMFT/dmft compared to patients with semisolid or solid diet [13], as liquid food usually has a higher cariogenic potential. Furthermore, oral clearance might be limited in patients suffering from oromotor dysfunction. In contrast, tube-fed patients were shown to have a lower caries experience as oral food intake is not possible [14, 15]. In the present study, caries experience and presence of periodontitis were not different among patients receiving pureed/liquid food, tube-fed patients, and patients without restrictions, assuming that nutrition is mainly indicating the level of disability.

Age and living situation showed a significant association to repeated GA only in the univariate analysis. Patients in need of dental treatment at younger age are probably more likely to need further dental treatment during the lifetime than patients that were older during the first treatment under GA. Interestingly, patients living alone or with family also tend to be at higher risk for repeated treatment. A recent study on oral health in older home care recipients and nursing home residents in Germany found poorer oral health in persons receiving home care [16]. The authors supposed that informal caregivers, such as relatives, are more likely to neglect oral hygiene or do not recognize non-painful changes of oral health [16]. In contrast, our study found a similar DMFT in patients living in a care facility (19.2 ± 7.7) and home care patients (16.9 ± 7.9), while the prevalence of periodontitis seemed higher in patients living in care facilities (94.4% vs. 78.1%). Therefore, it might also be speculated that informal caregivers of this vulnerable group of patients are more perceptive to dental treatment needs than institutional caregivers, leading to a slightly higher risk for repeated dental treatment under GA.

In conclusion, this retrospective study identified only few factors that might increase the risk for repeated dental treatment under GA in adult patients with intellectual and/or physical disabilities. More studies are needed to identify further risk factors for repeated dental treatment under GA.
